# Correction: Design and development of an irrigation monitoring and control system based on blynk internet of things and thingspeak

**DOI:** 10.1371/journal.pone.0326137

**Published:** 2025-06-10

**Authors:** Fahmy Rinanda Saputri, Ricardo Linelson, Muhammad Salehuddin, Danial Md Nor, Muhammad Imran Ahmad

In the published article [1], there is a data error in Table 4 and Table 6. The descriptions and conclusions based on these tables are also affected. Please see the correct Table 4 and Table 6 here.

**Table 4:** Air temperature measurement after calibration: hygrometer vs. DS18B20 sensor.

No.	Actual Hygrometer Temperature (°C)	DS18B20 Sensor Air Temperature Results (°C)					Mean (°C)	STD	Bias (°C)	Precision (%)	Accuracy (%)	Error (%)
		1	2	3	4	5						
1	30.7	30	30	30	30	30	30.0	0.0	0.0	100.00	97.72	2.28
2	30.7	30	30	30	30	30	30.0	0.0	0.0	100.00	97.72	2.28
3	30.7	30	30	30	30	30	30.0	0.0	0.0	100.00	97.72	2.28
4	30.7	30	30	30	30	30	30.0	0.0	0.0	100.00	97.72	2.28
5	30.5	30	30	30	30	30	30.0	0.0	0.0	100.00	98.36	1.64
Average									**0.7**	**100.0**	**97.8**	**2.2**

**Table 6:** Air humidity measurement: hygrometer vs. DHT11 sensor after calibration.

No.	Actual Hygrometer Humidity (%)	DHT11 Sensor Air Humidity Results (%)					Mean (%)	STD	Bias (%)	Precision (%)	Accuracy (%)	Error (%)
		1	2	3	4	5						
1	64	65	65	65	65	65	65.0	0.00	1.0	100.00	98.44	1.56
2	59	58	58	58	58	58	58.0	0.00	1.0	100.00	98.31	1.69
3	58	58	58	58	58	58	58.0	0.00	0.0	100.00	100.00	0.00
4	56	56	56	56	56	56	56.0	0.00	0.0	100.00	100.00	0.00
5	51	51	51	51	51	51	51.0	0.00	0.0	100.00	100.00	0.00
Average									**0.4**	**100.0**	**99.3**	**0.7**

Several errors were identified in the original analysis. The authors have provided the incorrect section (Before) alongside the revised and corrected version, with amendments highlighted in bold (After), as presented in the table here:

**Table:** 

Section	Before	After
**3.2 Air temperature measurement with DS18B20 sensor**	Table 3 presents the air temperature measurements obtained using the hygrometer and the DS18B20 sensor, including bias, precision, accuracy, and error values. The average bias for the DS18B20 sensor was −0.9°C, with a bias of −0.8, precision of 98.4%, accuracy of 96.9%, and error of 3.1%. High precision and accuracy indicate that the DS18B20 sensor delivers highly accurate results, with minimal deviation from the hygrometer used as a reference. Table 4 presents the results of the air temperature measurements using the hygrometer and DS18B20 sensor after calibration, including bias, precision, accuracy, and error. The average temperature measured by the DS18B20 sensor was 30.0°C with a standard deviation of 0, bias of 0.7°C, precision of 100%, accuracy of 97.8%, and error of 2.2%. The high precision and accuracy indicate that the DS18B20 sensor delivers excellent performance after calibration.	Table 3 presents the air temperature measurements obtained using the hygrometer and the DS18B20 sensor, including bias, precision, accuracy, and error values. **The average bias of the DS18B20 sensor before calibration was −0.8°C, with a precision of 98.4%, an accuracy of 96.9%, and an error rate of 3.1%.** The high precision and accuracy indicate that the DS18B20 sensor provides highly reliable results, with minimal deviation from the hygrometer used as the reference instrument. Table 4 presents the results of the air temperature measurements using the hygrometer and DS18B20 sensor after calibration, including bias, precision, accuracy, and error. **The DS18B20 sensor demonstrated improved performance, with an average bias of 0.7°C, a precision of 100.0%, an accuracy of 97.8%, and a reduced error rate of 2.2%.** The high precision and accuracy indicate that the DS18B20 sensor delivers excellent performance after calibration.
**3.3 Air humidity measurement with DHT11 sensor**	Table 5 displays the air humidity measurements obtained from the hygrometer and the DHT11 sensor, including bias, precision, accuracy, and error values. The DHT11 sensor exhibited an average bias of −0.2, precision of 98.8%, accuracy of 96.7%, and error of 3.3%. Table 6 shows the results of air humidity measurements obtained from the hygrometer and DHT11 sensor after calibration. The DHT11 sensor exhibited an average humidity of 65.0%, with a standard deviation of 0%, bias of 1.0%, precision of 100%, accuracy of 100%, and error of 0.8%. Despite the high precision and accuracy, minor variations with the hygrometer data were observed, indicating that the DHT11 sensor, while reliable, may require further monitoring. However, the study is limited by restricted field testing, and the system’s long-term reliability remains unverified. Future research should focus on extended field trials to assess durability and performance across diverse agricultural environments and climates.	Table 5 displays the air humidity measurements obtained from the hygrometer and the DHT11 sensor, including bias, precision, accuracy, and error values. The DHT11 sensor exhibited an average bias of −0.2%, precision of 98.8%, accuracy of 96.7%, and error of 3.3%. Table 6 shows the results of air humidity measurements obtained from the hygrometer and DHT11 sensor after calibration. **The DHT11 sensor demonstrated improved performance, with an average bias of 0.4%, a precision of 100.0%, an accuracy of 99.3%, and a reduced error rate of 0.7%.** These results indicate that the DHT11 sensor performs with excellent precision and high accuracy in measuring air humidity, with minimal deviation from the reference values. Despite the high precision and accuracy, minor variations with the hygrometer data were observed, indicating that the DHT11 sensor, while reliable, may require further monitoring. However, the study is limited by restricted field testing, and the system’s long-term reliability remains unverified. Future research should focus on extended field trials to assess durability and performance across diverse agricultural environments and climates.
