# Supplementary material for: Design and development of an irrigation monitoring and control system based on blynk internet of things and thingspeak
Source: PLoS One. 2025 Apr 17;20(4):e0321250. doi: 10.1371/journal.pone.0321250 (PMC12005828; doi:10.1371/journal.pone.0321250)
Supplement: S1 File — (DOCX) [file pone.0321250.s001.docx]

All relevant data required to replicate the findings of this study are included within the manuscript. The minimal data set, including values used for statistical analysis and measurements, has been provided to ensure full transparency and reproducibility.

**Table 1** Air temperature measurement: hygrometer vs. DS18B20 sensor

| No | Actual Hygrometer Temperature (°C) | DS18B20 Sensor Air Temperature Results (°C) | | | | | Mean (°C) | STD | Bias (°C) | Precision (%) | Accuracy (%) | Error (%) |
| --- | --- | --- | --- | --- | --- | --- | --- | --- | --- | --- | --- | --- |
|  |  | 1 | 2 | 3 | 4 | 5 |  |  |  |  |  |  |
| 1 | 31 | 31 | 31 | 32 | 32 | 32 | 31.6 | 0.5 | -0.6 | 98.45 | 97.19 | 2.81 |
| 2 | 38.2 | 38 | 39 | 39 | 39 | 40 | 39.0 | 0.6 | -0.8 | 98.38 | 97.13 | 2.87 |
| 3 | 42 | 42 | 43 | 43 | 44 | 44 | 43.2 | 0.7 | -1.2 | 98.27 | 97.51 | 2.49 |
| 4 | 45 | 44 | 45 | 46 | 46 | 46 | 45.4 | 0.8 | -0.4 | 98.24 | 95.56 | 4.44 |
| 5 | 48.3 | 48 | 49 | 49 | 50 | 50 | 49.2 | 0.7 | -0.9 | 98.48 | 97.22 | 2.78 |
| Average | | | | | | | | | -0.8 | 98.4 | 96.9 | 3.1 |

**Table 2** Air temperature measurement after calibration: hygrometer vs. DS18B20 sensor

| No | Actual Hygrometer Temperature (°C) | DS18B20 Sensor Air Temperature Results (°C) | | | | | Mean (°C) | STD | Bias (°C) | Precision (%) | Accuracy (%) | Error (%) |
| --- | --- | --- | --- | --- | --- | --- | --- | --- | --- | --- | --- | --- |
|  |  | 1 | 2 | 3 | 4 | 5 |  |  |  |  |  |  |
| 1 | 30.7 | 30 | 30 | 30 | 30 | 30 | 30.0 | 0.0 | 1 | 30.7 | 30 | 30 |
| 2 | 30.7 | 30 | 30 | 30 | 30 | 30 | 30.0 | 0.0 | 2 | 30.7 | 30 | 30 |
| 3 | 30.7 | 30 | 30 | 30 | 30 | 30 | 30.0 | 0.0 | 3 | 30.7 | 30 | 30 |
| 4 | 30.7 | 30 | 30 | 30 | 30 | 30 | 30.0 | 0.0 | 4 | 30.7 | 30 | 30 |
| 5 | 30.5 | 30 | 30 | 30 | 30 | 30 | 30.0 | 0.0 | 5 | 30.5 | 30 | 30 |
| Average | | | | | | | | | 0.7 | 100.0 | 97.8 | 2.2 |

**Table 3** Air humidity measurement: hygrometer vs. DHT11 sensor

| No | Actual Hygrometer Humidity (%) | DHT11 Sensor Air Humidity Results (%) | | | | | Mean (%) | STD | Bias (%) | Precision (%) | Accuracy (%) | Error (%) |
| --- | --- | --- | --- | --- | --- | --- | --- | --- | --- | --- | --- | --- |
|  |  | 1 | 2 | 3 | 4 | 5 |  |  |  |  |  |  |
| 1 | 61 | 60 | 61 | 61 | 62 | 62 | 61.2 | 0.75 | -0.2 | 98.78 | 96.65 | 3.35 |
| 2 | 60 | 59 | 60 | 61 | 61 | 61 | 60.4 | 0.80 | -0.4 | 98.68 | 96.67 | 3.33 |
| 3 | 60 | 59 | 59 | 60 | 60 | 61 | 59.8 | 0.75 | 0.2 | 98.75 | 95.93 | 4.07 |
| 4 | 60 | 60 | 60 | 61 | 61 | 61 | 60.6 | 0.49 | -0.6 | 99.19 | 98.55 | 1.45 |
| 5 | 59 | 58 | 58 | 59 | 59 | 60 | 58.8 | 0.75 | 0.2 | 98.73 | 95.86 | 4.14 |
| Average | | | | | | | | | -0.2 | 98.8 | 96.7 | 3.3 |

**Table 4** Air humidity measurement: hygrometer vs. DHT11 sensor after calibration

| No | Actual Hygrometer Humidity (%) | DHT11 Sensor Air Humidity Results (%) | | | | | Mean (%) | STD | Bias (%) | Precision (%) | Accuracy (%) | Error (%) |
| --- | --- | --- | --- | --- | --- | --- | --- | --- | --- | --- | --- | --- |
|  |  | 1 | 2 | 3 | 4 | 5 |  |  |  |  |  |  |
| 1 | 64 | 65 | 65 | 65 | 65 | 65 | 65.0 | 0.00 | 1 | 64 | 65 | 65 |
| 2 | 59 | 58 | 58 | 58 | 58 | 58 | 58.0 | 0.00 | 2 | 59 | 58 | 58 |
| 3 | 58 | 58 | 58 | 58 | 58 | 58 | 58.0 | 0.00 | 3 | 58 | 58 | 58 |
| 4 | 56 | 56 | 56 | 56 | 56 | 56 | 56.0 | 0.00 | 4 | 56 | 56 | 56 |
| 5 | 51 | 51 | 51 | 51 | 51 | 51 | 51.0 | 0.00 | 5 | 51 | 51 | 51 |
| Average | | | | | | | | | 0.0 | 100.0 | 100.0 | 0.0 |

**Table 5** Soil moisture (%) increase in response to water volume

| No | Water Volume (mL) | Capacitive Soil Moisture Sensor V2.0 Results (%) | | | | | Mean (%) | STD | Bias (%) | Precision (%) | Accuracy (%) | Error (%) |
| --- | --- | --- | --- | --- | --- | --- | --- | --- | --- | --- | --- | --- |
|  |  | 1 | 2 | 3 | 4 | 5 |  |  |  |  |  |  |
| 1 | 10 | 29 | 29 | 29 | 29 | 29 | 29.0 | 0.00 | -19.0 | 100.00 | 100.00 | 0.00 |
| 2 | 20 | 53 | 53 | 53 | 53 | 53 | 53.0 | 0.00 | -33.0 | 100.00 | 100.00 | 0.00 |
| 3 | 30 | 72 | 72 | 72 | 72 | 72 | 72.0 | 0.00 | -42.0 | 100.00 | 100.00 | 0.00 |
| 4 | 40 | 82 | 82 | 82 | 82 | 82 | 82.0 | 0.00 | -42.0 | 100.00 | 100.00 | 0.00 |
| 5 | 50 | 100 | 100 | 100 | 100 | 100 | 100.0 | 0.00 | -50.0 | 100.00 | 100.00 | 0.00 |
| Average | | | | | | | | | -37.2 | 100.0 | 100.0 | 0.0 |
