# Supplementary material for: Design and development of an irrigation monitoring and control system based on blynk internet of things and thingspeak
Source: PLoS One. 2025 Apr 17;20(4):e0321250. doi: 10.1371/journal.pone.0321250 (PMC12005828; doi:10.1371/journal.pone.0321250)
Supplement: Attachment — Submitted filename: Revisions needed.docx [file pone.0321250.s002.docx]

**Comments for Improvement of Manuscript PONE-D-24-54867**

**General Comments**

The manuscript discusses an IoT-based automatic irrigation system, demonstrating its potential in sustainable agriculture. The work is promising, but several areas need refinement to enhance clarity, technical rigor, and overall readability.

**Abstract**

1. Lines 15–31: The abstract provides a comprehensive overview but is overly dense. Simplify sentences and avoid technical details (e.g., sensor biases) here. Focus on the broader implications of the research.

For instance, the lines 15 – 31 can be simplified as follows “..............The scarcity of water resources exacerbated by climate change poses a major challenge for sustainable agriculture. This study presents an Internet of Things (IoT)-enabled irrigation system designed for real-time monitoring and precise water control. Using the Blynk platform and ThingSpeak for data management, the system integrates sensors for soil moisture, temperature, and humidity with a NodeMCU module to optimize irrigation practices. Initial results demonstrate the system’s effectiveness in improving water use efficiency and supporting sustainable agricultural practices, providing a low-cost, accessible solution for small and medium-scale farmers.........”

2. Line 33: Correct the keyword "water resourch" to "water resource."

**Introduction**

3. Lines 37–52: The introduction effectively sets the context but is repetitive about climate change and IoT benefits. Combine related sentences for conciseness.

What I mean here is that can we merge sentences discussing the impacts of climate change and the advantages of IoT systems to streamline the narrative. Focus on the unique contribution of integrating Blynk and ThingSpeak in this system, which sets it apart from previous studies.

4. Lines 56–61: Specify what distinguishes this study from previous works. Explicitly state the novel contributions (e.g., combination of Blynk and ThingSpeak).

e.g., Explain how this approach overcomes limitations of prior studies, such as the lack of integration between real-time monitoring and historical data storage. Emphasize that this integration provides farmers with both immediate feedback and long-term insights, addressing gaps in existing IoT-based irrigation solutions.

5. Lines 62–68: Ensure all acronyms are defined upon first use (e.g., NodeMCU V3).

**Materials and Methods**

6. Line 114: Define "mixed method approach" briefly for clarity.

What I expect here is to see the authors are improvising by stating like: “This research employs a mixed method approach, combining quantitative and qualitative methodologies. Quantitative methods involve real-time sensor data collection and analysis, while qualitative methods focus on observations and feedback from users during system implementation. This approach ensures a comprehensive understanding of both the technical performance and practical usability of the irrigation system." But this is just my opinion for clarity.

7. Lines 126–137: Expand Figure 1’s description. Mention the hardware/software components explicitly linked in the diagram.

Here we may provide a brief explanation of each component depicted, such as sensors, microcontrollers, and their roles in the system. Specify how these hardware and software elements interact to achieve real-time monitoring and irrigation control. Additionally, explain why these specific components were chosen over alternatives to strengthen the justification for the system design."

8. Lines 143–148: Provide a flowchart figure for the programming logic described here to help readers visualize the process.
9. Line 195: Clarify the "C++-based program" development phase by explaining the rationale for selecting C++ over other languages.
10. Lines 224–225: Explain why the relay module is chosen to control irrigation pumps—highlight its advantages.

**Results**

11. Line 312: Include labels for Figure 5’s components in the legend. This would help readers interpret the circuit design.
12. Line 374: Table 5 should clarify the unit for soil moisture (e.g., "%") in the title.
13. Lines 378–382: Mention the precision and accuracy of the soil moisture sensor in a separate subsection for consistency.

**Discussion**

14. Line 329: Discuss the broader implications of these findings for large-scale farming applications and if possible, to all types of farming scales.
15. Line 365: If necessary, address the limitations of the current study (e.g., restricted field testing or long-term reliability).
16. Lines 373–383: Expand on the strong linear relationship between water volume and soil moisture. Are there threshold effects?

**Conclusion**

17. Lines 384–402: Summarize the broader significance and practical applications of the research instead of repeating technical results already presented.
18. Lines 400–401: Suggest future additions (e.g., integration with machine learning for predictive analytics).

**References**

19. Lines 410–444: do the references follow the standardized formatting (that ensures all citations include complete details such as volume, issue, and DOI) if yes, its OK.

**Figures and Tables**

20. Figures 1–7: Enhance captions by providing additional context (e.g., what specific aspect each figure illustrates).
21. Table 1 and 2: Ensure consistent units and decimal formatting across tables.

**Language and Grammar**

22. Throughout: Check for typographical errors, such as "resourch" (Line 33) and "blynk" (capitalize consistently as "Blynk").
23. Throughout: Avoid overly technical jargon. Simplify where possible without losing technical accuracy.

**Additional Suggestions**

24. Include a cost analysis of the developed system to showcase its feasibility for smallholder farmers.
25. Add a subsection discussing the environmental benefits of the system compared to traditional irrigation methods.
