# Supplementary material for: Design and development of an irrigation monitoring and control system based on blynk internet of things and thingspeak
Source: PLoS One. 2025 Apr 17;20(4):e0321250. doi: 10.1371/journal.pone.0321250 (PMC12005828; doi:10.1371/journal.pone.0321250)
Supplement: Attachment — Submitted filename: Response to Reviewers.docx [file pone.0321250.s003.docx]

**Response to Comments of Improvement of Manuscript PONE-D-24-54867**

Dear Reviewers and Editors,

The authors of this paper greatly appreciate the valuable comments from the reviewers and editors. The suggestions are greatly helpful to improve the quality of our paper. We have made careful modifications strictly according to the Reviewer’s advice.

**General Comments**

The manuscript discusses an IoT-based automatic irrigation system, demonstrating its potential in sustainable agriculture. The work is promising, but several areas need refinement to enhance clarity, technical rigor, and overall readability.

**Abstract**

1. Lines 15–31: The abstract provides a comprehensive overview but is overly dense. Simplify sentences and avoid technical details (e.g., sensor biases) here. Focus on the broader implications of the research.

For instance, the lines 15 – 31 can be simplified as follows “..............The scarcity of water resources exacerbated by climate change poses a major challenge for sustainable agriculture. This study presents an Internet of Things (IoT)-enabled irrigation system designed for real-time monitoring and precise water control. Using the Blynk platform and ThingSpeak for data management, the system integrates sensors for soil moisture, temperature, and humidity with a NodeMCU module to optimize irrigation practices. Initial results demonstrate the system’s effectiveness in improving water use efficiency and supporting sustainable agricultural practices, providing a low-cost, accessible solution for small and medium-scale farmers.........”

**Response 1**: Thank you for the recommendation. Then it is the before and after revision of the abstract.

Before

The increasing scarcity of water resources, intensified by climate variability, poses a significant challenge to sustainable agriculture, particularly in regions with limited rainfall. Effective irrigation management is essential for optimizing water use, ensuring crop productivity, and conserving environmental resources. This study introduces an Internet of Things (IoT)-based automatic irrigation system aimed at real-time monitoring and precise water control, utilizing the Blynk platform and ThingSpeak database for data management. The system incorporates a DS18B20 temperature sensor, DHT11 humidity sensor, and soil moisture sensor, all integrated via a NodeMCU module for seamless connectivity. Initial testing demonstrated a precision of 98.4% and accuracy of 96.9% for the DS18B20 sensor, with a bias of -0.9°C, while the DHT11 sensor displayed a -0.2% average bias, 98.8% precision, 96.7% accuracy, and a 3.3% error margin. Despite its high precision, the DHT11 required further calibration to align with the reference hygrometer. Post-calibration, both sensors achieved 100% precision and accuracy, matching reference standards closely. Soil moisture analysis directly correlated with irrigation volume, where an additional 10 mL of water increased soil moisture by 29%. This IoT-enabled system provides farmers with enhanced control over irrigation, enabling data-driven decisions that promote efficient water use and sustainable agricultural practices

After

The scarcity of water resources exacerbated by climate change poses a major challenge for sustainable agriculture. This study presents an Internet of Things (IoT)-enabled irrigation system designed for real-time monitoring and precise water control. Using the Blynk platform and ThingSpeak for data management, the system integrates sensors for soil moisture, temperature, and humidity with a NodeMCU module to optimize irrigation practices. Initial results demonstrate the system’s effectiveness in improving water use efficiency and supporting sustainable agricultural practices, providing a low-cost, accessible solution for small and medium-scale farmers.

2. Line 33: Correct the keyword "water resourch" to "water resource."

**Response 2**: Yes, we have corrected the keyword. Thank you.

**Introduction**

3. Lines 37–52: The introduction effectively sets the context but is repetitive about climate change and IoT benefits. Combine related sentences for conciseness.

What I mean here is that can we merge sentences discussing the impacts of climate change and the advantages of IoT systems to streamline the narrative. Focus on the unique contribution of integrating Blynk and ThingSpeak in this system, which sets it apart from previous studies.

**Response 3**: This is the revised paragraph which has combined.

The increasing extremity of global climate change has created significant challenges for the agricultural sector, particularly in managing limited water resources [3], [4]. Rising temperatures and shifts in soil moisture patterns disrupt traditional irrigation methods, posing risks to crop yields. In response, Internet of Things (IoT)-based automatic irrigation systems have emerged as a promising solution, enabling real-time, demand-based watering guided by environmental data from sensors.The increasing extremity of global climate change has created significant challenges for the agricultural sector, particularly in managing limited water resources. Rising temperatures and shifts in soil moisture patterns disrupt the effectiveness of manual irrigation, posing risks to crop yields. In response, Internet of Things (IoT)-based automatic irrigation systems have emerged as a promising solution, enabling real-time, demand-based watering guided by environmental data collected from sensors [1], [2] [5], [6]. These systems enhance water use efficiency by utilizing sensors to monitor soil moisture, temperature, and other environmental factors that affect crop water requirements, providing smart solutions to address climate change challenges [7]. This study stands out by integrating Blynk for real-time control and ThingSpeak for cloud data storage, offering a more effective, accessible, and cost-efficient solution for farmers facing the impacts of climate change.

4. Lines 56–61: Specify what distinguishes this study from previous works. Explicitly state the novel contributions (e.g., combination of Blynk and ThingSpeak).

e.g., Explain how this approach overcomes limitations of prior studies, such as the lack of integration between real-time monitoring and historical data storage. Emphasize that this integration provides farmers with both immediate feedback and long-term insights, addressing gaps in existing IoT-based irrigation solutions.

**Response 4**: This is the novel of contribution that we have stated in the article.

This study addresses these gaps by integrating Blynk for real-time irrigation control and ThingSpeak for cloud-based data storage, enabling both immediate feedback and long-term insights. This combination provides farmers with enhanced control and the ability to analyze historical data to optimize irrigation practices and improve water efficiency, overcoming the limitations of previous systems.

5. Lines 62–68: Ensure all acronyms are defined upon first use (e.g., NodeMCU V3).

**Response 5**: Thank you for your reminder. We have made revision related to the acronyms. NodeMCU V3 is standing for Node Microcontroller Unit Version 3. We have also added the other acronyms.

**Materials and Methods**

6. Line 114: Define "mixed method approach" briefly for clarity.

What I expect here is to see the authors are improvising by stating like: “This research employs a mixed method approach, combining quantitative and qualitative methodologies. Quantitative methods involve real-time sensor data collection and analysis, while qualitative methods focus on observations and feedback from users during system implementation. This approach ensures a comprehensive understanding of both the technical performance and practical usability of the irrigation system." But this is just my opinion for clarity.

**Response 6**: Thank you for asking clarity. Here the explanation of our methods in this research.

This research employs a mixed method approach, combining quantitative and qualitative methodologies. The quantitative approach involves real-time data collection from sensors, including soil moisture, temperature, and air humidity, to evaluate the system's effectiveness in maintaining optimal land conditions. The qualitative approach is based on field observations and experiments conducted by researchers to assess the system's usability, flexibility, and practicality. This integrated approach ensures a comprehensive understanding of the system's performance in real-world scenarios.

7. Lines 126–137: Expand Figure 1’s description. Mention the hardware/software components explicitly linked in the diagram.

Here we may provide a brief explanation of each component depicted, such as sensors, microcontrollers, and their roles in the system. Specify how these hardware and software elements interact to achieve real-time monitoring and irrigation control. Additionally, explain why these specific components were chosen over alternatives to strengthen the justification for the system design."

**Response 7**: The addition paragraph for completely Figure 1’s description is:

Based on Figure 1, the automatic irrigation system in this study utilizes several key components, including NodeMCU as the main microcontroller with Wi-Fi connectivity to control the entire system and send data to the IoT platform. The sensors used include Capacitive Soil Moisture Sensor V2.0 to measure soil moisture, Temperature Waterproof Sensor DS18B20 to monitor the ambient temperature, and Humidity Sensor DHT11 to record air humidity. In addition, the system is equipped with a 5V relay module that controls the water pump, a 16x2 I2C LCD to display data locally, and a 5V/2A power supply as a power source. Additional components such as a 4.7 kΩ resistor are used to support communication with the DS18B20 sensor, while jumper cables connect all components in the system.

This automatic irrigation system works by collecting real-time data from soil moisture, temperature (DS18B20), and air humidity (DHT11) sensors, which are processed by NodeMCU as the main controller module. Based on the soil moisture data, the NodeMCU regulates the activation of the relay module to turn on the water pump automatically if the moisture is below a predetermined threshold. The system is equipped with a 16x2 LCD screen for local monitoring and uses the Blynk platform for real-time control via mobile devices, as well as ThingSpeak for cloud-based data storage and long-term analysis.

8. Lines 143–148: Provide a flowchart figure for the programming logic described here to help readers visualize the process.

**Response 8**: Thank you very much. The flowchart has been shown in the Figure 2 and 3.

9. Line 195: Clarify the "C++-based program" development phase by explaining the rationale for selecting C++ over other languages.

**Response 9**: The selection of the C++ language is based on the use of the Arduino IDE, which provides built-in libraries to support the integration of hardware such as sensors and relay modules. C++ enables efficient program development and makes it easy to test directly on the microcontroller, making it ideal for IoT applications.

10. Lines 224–225: Explain why the relay module is chosen to control irrigation pumps—highlight its advantages.

**Response 10**: Ok, we have explained it. The relay module is chosen to control irrigation pumps because it provides a reliable and efficient way to interface low-power microcontrollers, such as NodeMCU, with high-power devices like water pumps. It acts as an electrically isolated switch, ensuring safe operation by preventing direct current surges from reaching the microcontroller.

**Results**

11. Line 312: Include labels for Figure 5’s components in the legend. This would help readers interpret the circuit design.

**Response 11**: We have made revision in this figure.

12. Line 374: Table 5 should clarify the unit for soil moisture (e.g., "%") in the title.

**Response 12**: Yes, we have added it. Thank you.

13. Lines 378–382: Mention the precision and accuracy of the soil moisture sensor in a separate subsection for consistency.

**Response 13**: Here the addition sentence.

The V2.0 Capacitive Soil Moisture Sensor demonstrated excellent precision and accuracy during testing. Precision, which is calculated based on repeated measurements under consistent conditions, consistently came in at 100%, indicating highly reliable readings. In addition, the accuracy of the sensor reached 100%, ensuring that the measured value was in line with the actual soil moisture level. These results confirm the suitability of the sensor for precise monitoring in automated irrigation systems.

**Discussion**

14. Line 329: Discuss the broader implications of these findings for large-scale farming applications and if possible, to all types of farming scales.

**Response 14**: Thank you, we have made new paragraph related to this.

The findings of this research provide the potential for IoT-based irrigation systems to revolutionize water management across different scales of agriculture. For large-scale farms, these systems offer significant advantages by automating the irrigation process and enabling real-time monitoring over large areas. This level of precision can optimize water use, minimize wastage, and increase crop yields, while reducing labor and operational costs associated with traditional irrigation methods. The ability to collect and analyze data continuously allows farm managers to make informed decisions, improving overall efficiency and sustainability.

On smaller farms, the system provides a cost-effective solution that empowers farmers to manage water resources more efficiently. Its modular and scalable design ensures that it can be customized to meet the needs of different farm sizes and types, whether for subsistence farming or smallholder operations. By making advanced technology accessible to all, the system encourages inclusiveness in adopting sustainable practices, contributing to long-term agricultural resilience.

15. Line 365: If necessary, address the limitations of the current study (e.g., restricted field testing or long-term reliability).

**Response 15**: The addition explanation is However, the study is limited by restricted field testing, and the system's long-term reliability remains unverified. Future research should focus on extended field trials to assess durability and performance across diverse agricultural environments and climates.

16. Lines 373–383: Expand on the strong linear relationship between water volume and soil moisture. Are there threshold effects?

**Response 16**: The strong linear relationship between water volume and soil moisture is evident from the data in Table 3, where an increase in the injected water volume consistently leads to a corresponding increase in soil moisture, measured by the Capacitive Soil Moisture Sensor V2.0. This relationship demonstrates the predictive and direct effect that the volume of water has on the moisture content of the soil. For instance, 10 mL of water results in a 29% increase in moisture, while 50 mL achieves a maximum of 100% moisture, showing that the system can reliably measure and react to changes in moisture levels.

However, the data also suggests the presence of threshold effects at higher water volumes. As the water volume increases beyond 40 mL, the increase in soil moisture becomes less pronounced, reaching 100% moisture with 50 mL. This diminishing return implies that there is a point at which adding more water does not significantly improve soil moisture content. Once the soil reaches saturation, additional water may not be effectively absorbed, potentially leading to waterlogging, which can be detrimental to plant health by reducing root oxygenation and promoting anaerobic conditions.

**Conclusion**

17. Lines 384–402: Summarize the broader significance and practical applications of the research instead of repeating technical results already presented.

**Response 17**: Here the addition.

Future improvements could include integrating machine learning for predictive analytics to further enhance irrigation efficiency by anticipating water needs based on weather patterns and soil conditions. Additionally, expanding the system to include other environmental sensors or integrating it with broader smart farming technologies could offer a comprehensive solution for sustainable agricultural management.

18. Lines 400–401: Suggest future additions (e.g., integration with machine learning for predictive analytics).

**Response 18**: Here the addition.

Future improvements could include integrating machine learning for predictive analytics to further enhance irrigation efficiency by anticipating water needs based on weather patterns and soil conditions. Additionally, expanding the system to include other environmental sensors or integrating it with broader smart farming technologies could offer a comprehensive solution for sustainable agricultural management.

**References**

19. Lines 410–444: do the references follow the standardized formatting (that ensures all citations include complete details such as volume, issue, and DOI) if yes, its OK.

**Response 19**: It is automatically formed because we use Mendeley for arranging the references.

**Figures and Tables**

20. Figures 1–7: Enhance captions by providing additional context (e.g., what specific aspect each figure illustrates).

**Response 20**: we have added legend in the Figure 5.

21. Table 1 and 2: Ensure consistent units and decimal formatting across tables.

**Response 21**: Unit for mean and bias are based on the measured parameter.

**Language and Grammar**

22. Throughout: Check for typographical errors, such as "resourch" (Line 33) and "blynk" (capitalize consistently as "Blynk").

**Response 22**: We have checked and replace it, but for that term in the title still using not capitalize. How about it?

23. Throughout: Avoid overly technical jargon. Simplify where possible without losing technical accuracy.

**Response 23**: Thank you very much for your reminding.

**Additional Suggestions**

24. Include a cost analysis of the developed system to showcase its feasibility for smallholder farmers.
25. Add a subsection discussing the environmental benefits of the system compared to traditional irrigation methods.

**Responses 24 and 25**: Thank you for the suggestions. We have added the new part before conclusion.

4.5 Feasibility and Broader Implications of IoT-Based Automatic Irrigation Systems

A cost analysis was conducted to assess the feasibility of implementing an IoT-based automatic irrigation system for smallholder farmers. This system, built using a NodeMCU microcontroller, DS18B20 temperature sensor, DHT11 humidity sensor, soil moisture sensor, relay module, DC pump, LCD display, and additional peripherals, has a hardware cost of IDR 235,000 (~USD 15.00). The system operates on a 5V USB power source with an average power consumption of 1.0W, resulting in minimal operational costs. Daily energy consumption is 24Wh or 0.024kWh, calculated as 1.0W × 24 hours. Based on an electricity rate of IDR 1,352/kWh, the daily electricity cost amounts to IDR 32.45. Over a year, this translates to an annual electricity cost of approximately IDR 11,843.52 with a calculation of IDR 32.45/day × 365 days.

When compared to traditional irrigation, which requires 1000mL of water per plant, twice a day, this IoT-based system reduces water usage by 30% through real-time monitoring and optimization of irrigation based on soil moisture levels. By maintaining soil moisture within the ideal range of 30%-60%, the system prevents overwatering and minimizes water wastage, promoting healthier plant growth. The soil moisture levels are categorized as dry (0%-30%), moist (30%-60%), and wet (60%-100%), and the system continuously monitors these conditions using sensors to ensure optimal irrigation.

This IoT-based solution is particularly impactful for smallholder farmers, offering a scalable and cost-effective approach to sustainable water management. The modular design allows for customization to suit varying farm sizes and types, making it adaptable for subsistence farming or small-scale agricultural operations. By reducing water usage and increasing irrigation efficiency, the system promotes sustainability while supporting improved crop productivity.

For larger agricultural operations, the benefits are equally compelling. The system’s ability to automate irrigation processes and provide real-time monitoring across expansive areas optimizes water use and minimizes wastage. This level of precision can significantly enhance crop yields while reducing labor and operational costs associated with traditional irrigation methods. Additionally, continuous data collection and analysis enable informed decision-making, further improving farm management efficiency and sustainability.

The broader implications of adopting IoT-based irrigation systems are transformative. By making advanced technology accessible and adaptable for farmers of all scales, these systems encourage the widespread adoption of sustainable practices. The resulting improvements in water efficiency and crop productivity contribute to long-term agricultural resilience, fostering food security and environmental sustainability across diverse farming contexts. This aligns directly with Sustainable Development Goals (SDGs), particularly Goal 2 (Zero Hunger), Goal 6 (Clean Water and Sanitation), and Goal 12 (Responsible Consumption and Production). By addressing these goals, IoT-based irrigation systems support the creation of resilient agricultural systems that can adapt to climate change, ensure efficient resource use, and provide equitable access to technological advancements for all farmers.
